# *Arisaema heterophyllum Blume* Monomer Stigmasterol Targets PPAR*γ* and Inhibits the Viability and Tumorigenicity of Lung Adenocarcinoma Cells NCI-H1975

**DOI:** 10.1155/2022/5377690

**Published:** 2022-07-20

**Authors:** Na Song, Jing Wang, Zonglang Lai, Shuting Liang, Wenjuan Zou, Juan Wang, Dandan Zheng, Ying Li, Yuxi He, Jun Cheng, Yue Wu

**Affiliations:** ^1^Department of Oncolology, Chongqing Hospital of Traditional Chinese Medicine, Chongqing 400020, China; ^2^College of Chemical and Environmental Engineering, Chongqing University of Arts and Sciences, Chongqing 402160, China; ^3^Department of Nephrology, Chongqing Hospital of Traditional Chinese Medicine, Chongqing 400020, China; ^4^Department of Gastroenterology, Chongqing Hospital of Traditional Chinese Medicine, Chongqing 400020, China

## Abstract

To clarify the regulatory effect and molecular mechanism of *Arisaema heterophyllum Blume* (AhBl) monomer stigmasterol on lung adenocarcinoma in human lung adenocarcinoma cells NCI-H1975 cultured *in vitro* and in nude mice. Oil red O staining, free fatty acid detection, adenosine triphosphate (ATP), and NADPH were applied to elucidate the regulatory effect of stigmasterol on the energy metabolism of NCI-H1975 cells. Simultaneously, colony formation assay and nude mouse tumorigenesis were performed to clarify the underlying mechanisms of stigmasterol on the proliferation and tumorigenesis of NCI-H1975 cells. Furthermore, peroxisome proliferator-activated receptor gamma (PPAR*γ*) inhibitor GW9662 was supplemented to determine the expression changes of cyclins to clarify the regulation mechanism of stigmasterol. The results revealed that stigmasterol administration markedly inhibited the viability but promoted lipid deposition of NCI-H1975 cells. Meanwhile, the reduction of cell energy metabolism affected cell proliferation and colony formation. qPCR and western blot assays indicated that stigmasterol played a role in regulating the expression of cyclins and PPAR*γ* signaling pathway proteins. Nude mouse tumorigenesis suggested that tumor size and weight in the stigmasterol-treated group were apparently lower as compared with the control group. Tumor tissue cells developed varying degrees of degeneration and large areas of ischemic necrosis presented in the central and peripheral cells. Immunohistochemistry results revealed that Ki67 expression in the stigmasterol group was substantially inhibited, while PPAR*γ* expression was greatly elevated as compared with the control. GW9662 could mediate the inhibitory effect of stigmasterol on NCI-H1975 cells. The current study demonstrated that stigmasterol targeted PPAR*γ* and inhibited the viability and tumorigenicity of lung adenocarcinoma cells NCI-H1975.

## 1. Introduction

Medicinal plants represent the oldest form of medicine and have been utilized for thousands of years in many countries. It is estimated that up to 75% of the population, particularly those living in developing countries, depend on plants as a source of folk medicine to meet their primary health care needs [1]. The World Health Organization (WHO) has recorded 21,000 plants with medicinal value out of 300,000 species available worldwide [2]. Medicinal plants are the main sources of various bioactive substances including polysaccharides, glycosides, alkaloids, polyphenols, coumarins [3], etc. The medicinal health functions and therapeutic effects of plant bioactive substances are mainly attributed to their various biological and pharmaceutical effects, such as antioxidant, antibacterial, antivirotic, anti-inflammatory, antineoplastic, and antidiabetic activities [4, 5]. The utilization of drugs of plant origin to treat multiple humankind diseases are less harmful and cheaper than modern drugs. The large-scale production of medicinal plants and their derivatives is an inevitable development tendency.

Lung cancer currently ranks the top mortality among global malignant tumors, and rates of metastasis and recurrence of advanced lung cancer remain at a high level [6]. According to the Global cancer statistics, in 2020, there were 11.4% (2.17/19.3 million) new lung cancer cases and 18% (1.8/10 million) lung cancer deaths worldwide [7]. The metastasis rate of lung cancer reaches 93% and the major sites of metastasis include the liver, brain, kidney, bone, etc. [8]. In addition, the recurrence rates of lung cancer patients in TNM stages I, II, and III are 34, 55, and 74%, respectively [9]. Unfortunately, most advanced lung cancer victims have no access to purchase drugs available. It is therefore that new anti-lung cancer drugs are still urgently needed.

In China, medicinal plants are also known as Chinese medicinal herbs or traditional Chinese medicine (TCM), which are well-recognized as one of the traditional treasures of China with a long history and have been extensively applied. With the advancement of modern medical technology, more in-depth research on Chinese medicinal herbs has been carried out. Numerous reports have demonstrated the antitumor effect of active monomer components of TCM at home and abroad [10]. Because Chinese medicinal herbs have the advantages of long-term action, multiple targets, low toxicity, and few side effects, they can effectively increase the cure rate of tumors and patients can benefit from a better quality of life. Therefore, it is of great importance to conduct research on the regulation and drug action mechanisms of Chinese medicinal herbs for lung cancer, from which patients can benefit much from the process of disease treatment.

“To fight poison with poison” therapy has been written in Volume 29 of the “Chuogenglu” (Retirement to the countryside) by Tao Zongyi from the Ming Dynasty, through which the poison can be detoxified. That is, toxic drugs can be applied to treat diseases caused by poison. Although disease conditions are complicated and vary constantly, the invasion of evil poison is the pathogenic cause of the disease. As the poison must be detoxified, only strong and powerful drugs in nature can be applied to restrict the poison, which is the commonly used method of “fighting poison with poison.” Modern medical research has also proved that the anticancer mechanism of toxic Chinese medicinal herbs mainly exerts a cytotoxic effect by directly killing cancer cells, by inducing apoptosis and differentiation [11]. For example, the modern pharmacology of the poisonous Chinese medicinal herb *Arisaema heterophyllum Blume* (AhBl) has been confirmed by Zheng Weiqin, a famous senior Chinese medicine expert that the water extract of AhBl has a specific inhibitory effect on cancer cells. Compound Sansheng injection containing AhBl produces an obvious inhibitory effect on lung cancer and Ehrlich ascites carcinoma in mice [12]. Stigmasterol represents a typical phytosterol, and it is widely present in the deodorized distillate of various plants and plant oil refining. The content accounts for about 22%. The monomer can be obtained through physical purification, characterized by rich nutrition, and strong physiological activity [13]. This material has been widely applied in the biology and medicine fields. Recent research has reported that stigmasterol exerts multiple pharmacological effects including antitumor, blood cholesterol reduction, and anti-osteoarthritis [14–16].

Peroxisome proliferators activated receptor gamma (PPAR*γ*) belongs to the nuclear hormone receptor superfamily, which is mainly expressed in adipose tissues as well as the immune system, and its affinity to the adipose-specific peroxidase proliferator response unit is higher than that of the other two isoforms. PPAR*γ* produces vital significance in multiple signaling pathways as it is the intersection of various signaling pathways in the human body, PPAR*γ* has various biological functions, mainly including regulating lipid metabolism and glucose metabolism, inhibiting tumor angiogenesis, invasion, metastasis, and inflammatory response, and inducing tumor cell differentiation and apoptosis [17]. Normally, the activation of PPAR*γ* requires the binding of corresponding ligands. After being activated, PPAR*γ* binds to the peroxisome proliferator responsive element (PPRE), thereby being responsible for corresponding transcription and regulation. Studies have shown that it can regulate the expressions of multiple downstream growth-regulating genes c-jun, ras, and c-myc [18]. In addition to regulating its downstream genes, PPAR*γ* can also mediate expressions of urokinase-type fibrinolysis zymogen activators, adhesion molecules, cytokines, and signal transduction molecule NF-*κ*B associated with multiple disease occurrence and development [19–21]. Many genes and factors regulated by PPAR*γ* are also intimately correlated with tumor formation and development.

Based on previous studies, this study put forward the hypothesis that stigmasterol, the main drug monomer of AhBl, targeted PPAR*γ* and inhibited the viability and tumorigenicity of lung adenocarcinoma cells NCI-H1975. This study intends to carry out both animal experiments and cell experiments applying modern advanced detection approaches to verify whether stigmasterol produces any effects on cell viability and tumorigenicity of lung adenocarcinoma cell NCI-H1975. By detecting the expression of cyclin and PPAR*γ*, we further explored the molecular mechanisms of stigmasterol against lung adenocarcinoma. Meanwhile, the scientific connotation of TCM “fighting poison with poison” was illustrated for lung cancer prevention and management, expecting to offer new ideas and directions for lung cancer therapies using TCM.

## 2. Materials and Methods

### 2.1. Network Pharmacology Analysis and Molecular Docking

The main components of AhBl were obtained from the Traditional Chinese Medicine Systems Pharmacology Database and Analysis Platform (TCMSP) (https://lsp.nwu.edu.cn/tcmsp.php). Main active ingredients were sorted out under the conditions of oral bioavailability (OB) ≥30% and drug likeliness (DL) ≥0.18. PharmMapper was adopted to obtain the possible target genes of each active ingredient. VCI disease targets were obtained from the disease database Genecards (https://auth.lifemapsc.com/) and a Venn diagram was subsequently plotted using the collected targets. Cytoscape software (version 3.7.1) was adopted to plot a diagram of the interaction network between drugs and targets. The molecular structures of stigmasterol and PPAR*γ* were exported from PubChem (https://pubchem.ncbi.nlm.nih.gov/) and the PDB database (https://www.rcsb.org), respectively, which were for subsequent molecular docking using Autodock software.

### 2.2. Cell Culture

NCI-H1975 cells were purchased from the Shanghai Advanced Research Institute of Chinese Academy of Sciences and then cultured in DMEM medium (BasalMedia, S310KJ, China) containing 10% FBS (Life-iLab, AC03L055, China) and 1% penicillin-streptomycin (Beyotime, C0009) under conventional culture conditions (5% CO_2_, 37°C).

### 2.3. MTT Assay

Stigmasterol was provided by Dr. Shuting Liang of the College of Chemical and Environmental Engineering, Chongqing University of Arts and Sciences, Chongqing, China. NCI-H1975 cells were grouped as follows: the cells were added to 500, 100, 20, 4, 0.8, and 0.16 *μ*mol/L of stigmasterol culture medium, 100 *μ*L each well, respectively. Three replicate wells were set up for each concentration. Following culture for 24 h, an MTT assay was performed, and the cell inhibition rate was calculated based on the measured optical density (OD) values at 570 nm using a microplate reader (Multiskan FC, ThermoFisherScientific, USA).

Each well of a 96-well plate was planted with 200 *μ*L of cells, with three multiple pores in each group. The 96-well plates were transferred and cultured in an incubator (37°C, 5% CO_2_). When cell adherence occurred, the cells were grouped as per the previously described cell modeling and subsequently kept in a 5% CO_2_ incubator. When the cells were incubated for 24 h, the 96-well plates were removed, supplied with 20 *μ*L 5 mg/mL of MTT solution (Solarbio Life Sciences, M1020, China) to each well, and cultured for 4 h in the incubator. The culture was then terminated, and the medium in the well was carefully aspirated. After adding 150 *μ*L dimethyl sulfoxide to each well, the cells were placed in a shaker and vibrated slowly for 10 min until the crystals were fully dissolved. The absorbance was determined at OD490 nm using an enzyme-linked immunometric meter (ELx800, Bio-Tek, USA).

### 2.4. Detection of Cellular Lipid Deposition using Oil Red O Staining

Following the NCI-H1975 cell culture, the cells were divided into three groups: control, low stigmasterol concentration (20 *μ*mol/L), and high stigmasterol concentration (100 *μ*mol/L).

After the removal of the used medium 24 h later, the cells followed two cycles of washing using distilled water. By supplementing with 4% paraformaldehyde, the cells were fixed for 30 min at room temperature. The solution was abandoned and followed by three cycles of washing using distilled water, the cells were immersed in 60% isopropanol for 5 min. Following the removal of the isopropanol, oil red O working solution (Solarbio Life Sciences, G1260, China) was added for staining for 20 min and discarded subsequently. After two cycles of washing with distilled water, there was no excess staining solution left and the cells were covered with distilled water and observed under a microscope (MF53-N, Mingmei, China).

### 2.5. Determination of Free Fatty Acid Content

The free fatty acid detection test was carried out with reference to the kit instructions (Solarbio Life Sciences, BC0590, China). The well-grown cells were collected by removal of used medium, washed with PBS once, planted in a six-well plate, by supplementing 200 *μ*L of western and IP cell lysis buffer to each well, lysed for 5 s, and collected using an appropriate centrifuge tube after lysis. The cells were centrifuged at 10,000*g* for 10 min to collect the supernatant for the determination.

### 2.6. Determination of ATP Content

The cells were collected into a centrifuge tube. The supernatant was discarded, and 1 mL of the extract was provided for ultrasonication 1 min and centrifuged at 10,000*g* at 4°C for 10 min. The supernatant was transferred to another centrifuge tube, and 500 *μ*L of chloroform was added, blended evenly, and centrifuged at 10,000*g* at 4°C for 3 min. When collected, the supernatant was placed on ice for testing. After being diluted 16 times with distilled water, 10 *μ*mol/mL ATP standard solution (Solarbio Life Sciences, BC0305, China) was set at 0.625 *μ*mol/mL standard solution for later use. After adding and mixing the sample to be tested, the OD value A1 at 340 nm for 10 s was measured immediately. The reaction continued for 3 min at 37°C incubator, and the OD value A2 at 3 min 10 s was measured.

### 2.7. Determination of NADPH Content

Subsequently, 200 *μ*L of NADP+/NADPH extract solution (Beyotime, S0179, China) was added to the cells and lysed for 10 min. After centrifugation at 12,000*g* at 4°C for 5 min, the supernatant was used as the sample to be tested. The 1 mM NADPH standard was diluted to the concentrations of 0, 0.25, 0.5, 1, 2, 4, 6, and 8 *μ*M using NADP+/NADPH extract. At determination, each well of the 96-well plate was provided with 50 *μ*L standard, equivalent to 0, 12.5, 25, 50, 100, 200, 300, and 400 pmol of NADPH per well. The point at a concentration of 0 *μ*M was the blank control point, containing only NADP+/NADPH extract. Of 100 *μ*L sample to be tested was aspirated to a centrifuge tube using a pipette and heated in a water bath at 60°C for 30 min for NADP+ decomposition. After the addition of 100 *μ*L of working solution for detection, each well was covered with a film and incubated at 37°C for 30 min. The plate was spun dry after the removal of the solution and followed by five cycles of washing. A film was applied to cover the microplate by supplementing each well with 90 *μ*L substrate solution, and development was carried out at 37°C for 10 min away from the light. The reaction was terminated after the wells were added with 50 *μ*L stop solution, each OD value was read at 450 nm using a microplate reader.

### 2.8. Cell Colony Formation Assay

NCI-H1975 cells at the logarithmic growth stage were taken for the preparation of cell suspension and inoculated at 800 cells/well in the six-well plates. According to the experimental grouping, each group had three multiple wells and was cultured for 3 weeks. When visible colony mass appeared in the Petri dish, cell culture was terminated. The supernatant was discarded, 4% paraformaldehyde (Leagene Biotech, DF0135, China) was applied for fixation for 20 min, followed by crystal violet staining (Leagene Biotech, DZ0053, China) for 15 min. The colony formation rate is calculated by (Number of clones/Number of inoculated cells) × 100%.

### 2.9. Detection of Gene Expression by qPCR

Trizol method was employed to extract total RNA, and 2 × T5 Fast qPCR Mix (SYBR Green I) was applied for the qPCR test. The reaction system included 2 × T5 Fast qPCR Mix, 10.0 *μ*L; 10 *μ*M Primer F, 0.8 *μ*L; 10 *μ*M Primer R, 0.8 *μ*L; 50x ROX Reference Dye II, 0.4 *μ*L; Template DNA, 0.5 *μ*L; and ddH_2_O, 7.5 *μ*L. Reaction conditions include 95°C, 30 s; 95°C, 5 s; 55°C, 30 s; and 72°C, 30 s, 40 cycles in total. Primer sequences including cyclinD1, CDK2, CDK4, CDK6, p21, PPAR*γ*, SIRT1, and GAPDH were shown in Table 1. The primers were synthesized by Beijing General Biomedicine Biotechnology Co. Ltd.

### 2.10. Protein Expression by Western Blot

After cell centrifugation, precooled RIPA lysate (Beyotime, P0013B, China) was added for lysis on ice for 15 min, vortex shaking for 30 s, and lysed on ice for another 15 min. After centrifugation at 12 000 r/min for 10 min, the supernatant was collected. The samples were quantified using bicinchoninic acid (BCA) kit (Beyotime, P0012, China) and the final protein concentration was set at 2.5 *μ*g/*μ*L by adding PBS and loading buffer. The protein was denatured first, incubated at 100°C for 5 min, and kept in a −80°C in a refrigerator. By adding 10% SDS-PAGE gel, electrophoresis was conducted at 80 and 120 V constant voltage for 30 and 90 min, respectively. The wet transfer was conducted under a 70 V constant voltage for 90 min. After membrane transfer, TBST solution containing 5% skim milk powder was added for membrane sealing at room temperature for 1 h. Primary antibodies were applied for incubation at 4°C in a slow shaker overnight. The next day, TBST was employed for film washing 3 times, 10 min each. After 2 h of incubation with secondary antibodies, repeated incubation was performed by adding ultrasensitive luminescence solution for 1 min and color development using a gel imaging system. Gray value analysis was based on ImageJ (Rawak Software, Germany). The primary antibodies of anti-cyclinD1 (A19038), CDK2 (A0294), CDK4 (A11136), CDK6 (A19038), p21 (A19038), acetyl-p53 (A19038), PPAR*γ* (A19038), SIRT1 (A19038), p-SIRT1 A19038, and *β*-actin (A19038) were purchased from ABclonal Technology, China.

### 2.11. Nude Mouse Tumorigenicity Assay

Experimental animals: a total of 15 male SPF grade nude mice (4–6 weeks) weighing approximately 15–20 g were from Chongqing Ensiweier Biotechnology Co. Ltd. (Animal License number (SCXK (Xiang) 2019–0004)). Fifteen 3-week-old nude mice were raised in animal houses, under the condition of 12 h light-dark cycles at 23–25°C, and supplied with food and water ad libitum. Meanwhile, NCI-H1975 cells were collected at the logarithmic growth stage, resuspended to 3 × 10^7^/mL in a basal medium free from serum, primary, and secondary antibodies, and prepared into cell suspension for tumorigenicity. After 7 days of adaptive feeding, the nude mice were inoculated subcutaneously in the right axilla near the dorsal waist at 100 *μ*L (about 3 × 10^6^ cells/mouse) dosage. When the tumor size was measured at 3 × 3 mm (around 12 d), the tumor-bearing nude mice that met the standard were grouped into control and stigmasterol groups randomly, with 5 mice in each group. Stigmasterol (40 mg/kg) was given intragastrically to the treatment group and normal saline for the control group each day. Tumor volume was measured every 2 d from the start of administration. The animals were sacrificed 20 d after administration, the tumor was weighed and cleaned with PBS, and fixed with paraformaldehyde for later use.

### 2.12. HE Staining

The specimens were collected, washed using PBS, followed by fixation with 4% paraformaldehyde, and soaked in 70, 80, 90, 95, and 100% ethanol for 1.5 h, respectively. After dehydration, the samples were treated for transparency using xylene for 20 min and dipped in melted paraffin for 3 h to prepare 2.5-*μ*m-thickness sections, which were subsequently heated in an oven at 55°C, ensuring the tissue sections to stick tightly to the adherent slides. Following transparency, dewaxing, and dehydration, the sections were washed thrice with double distilled water for 2 min each, stained with hematoxylin (Servicebio, G1004, China) for 3 min, and rinsed the excess staining solution with tap water for about 3 min. The sections were destained using 1% hydrochloric acid alcohol to remove excess hematoxylin staining solution in the cytoplasm, followed by eosin staining for 60 s. The excess dye solution was rinsed using tap water for about 3 min. Dehydration was performed using 75% ethanol for 10 s, 95% ethanol for 10 s, and finally anhydrous ethanol for 1 min. Xylene was employed for transparency for 5 min, sealed using neutral resin (Sinopharm, 10004160, China), and detected under a microscope. The nucleus was displayed in blue and the cytoplasm was red or pink. Mshot MF53 microscope from Guangzhou Mingmei Optoelectronic Technology Co. Ltd was utilized for photography.

### 2.13. Immunohistochemistry

The samples were cleaned using PBS, sectioned, sterilized by adding 3% H_2_O_2_, and incubated for 15 min at room temperature. By supplementing goat serum (Beyotime, C0265, China), the sections were blocked for 60 min at room temperature, taken out, and dried. A primary antibody was provided and incubated at 4°C overnight (ki67, A2094, 1:200, ABclonal, China; PPAR*γ*, A0270, 1:200, ABclonal, China). The specimens were washed thrice using PBS for 5 min. A secondary antibody working solution was used for incubation at room temperature for 1.5 h and washed again as previously described. DAB (ZSGB-BIO, ZLI-9019, China) was applied for development for 10 min (in the dark). Hematoxylin was used for restaining 5 min, followed by 1% hydrochloric acid ethanol differentiation for 5 s, and 95% ethanol dehydration for 2 min. Fresh 95% ethanol was employed for dehydration for another 2 min, and xylene was employed for transparency for 5 min. Fresh xylene was used for transparency for an additional 5 min, and the slides were fixed with neutral resin. The sections were photographed by an inverted Mshot MF53 microscope produced by Guangzhou Mingmei Optoelectronic Technology Co. Ltd.

### 2.14. Cell Treatment with PPAR*γ* Inhibitor

The PPAR*γ* inhibitor GW9662 (HY-16578, CAS No. 22978-25-2) was bought from MedChemExpress, USA. Following the NCI-H1975 cell culture, the cells were classified into control, high-concentration stigmasterol (100 *μ*mol/L), high-concentration stigmasterol + low-concentration GW9662 (20 *μ*mol/L), and high-concentration stigmasterol + high-concentration GW9662 (40 *μ*mol/L) groups. Follow-up experiments were conducted according to the grouping.

### 2.15. Statistical Analysis

Data statistical software was Graphpad Prism 8.0 (Graphpad, USA). ANOVA method was employed for data statistics and analysis. All measurement data were expressed by mean ± SEM. The value of *p* < 0.05 was considered statistically significant.

## 3. Results

### 3.1. Stigmasterol as a Primary Active Component of AhBl

**Figure 1:**
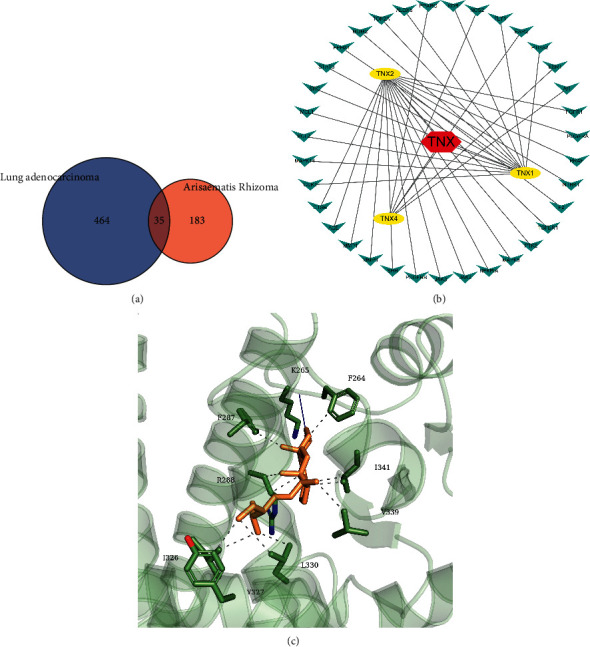
Network pharmacology and molecular docking analyzes the regulatory relationship between AhBl-stigmasterol-lung adenocarcinoma-PPAR*γ*. (a) Venn diagram of AhBl drug targets and lung adenocarcinoma-related targets. (b) Network pharmacological interaction diagram. (c) Molecular docking results. Stigmasterol is the main active component of AhBl. TNX indicates AhBl; TNX1 is 8,11,14-docosatrienoic acid, methyl ester; TNX2 is [(2R)-2-[[[(2R)-2-(benzoylamino)-3-phenylpropanoyl]amino]methyl]-3-phenylpropyl] acetate; TNX4 is sitosterol.

Based on network pharmacology, the active components and relevant targets of AhBl were retrieved from TCMSP, and lung adenocarcinoma-related genes were sorted out from the Genecards disease database (Figure 1(a)). Some target proteins of stigmasterol overlapped with disease targets of lung adenocarcinoma as presented in the network diagram of drug-active components-target genes (Figure 1(b)). Molecular docking prediction results indicated that stigmasterol and PPAR*γ* had strong binding interactions (Figure 1(c)).

### 3.2. Stigmasterol Inhibits NCI-H1975 Cell Viability and Energy Metabolism

**Figure 2:**
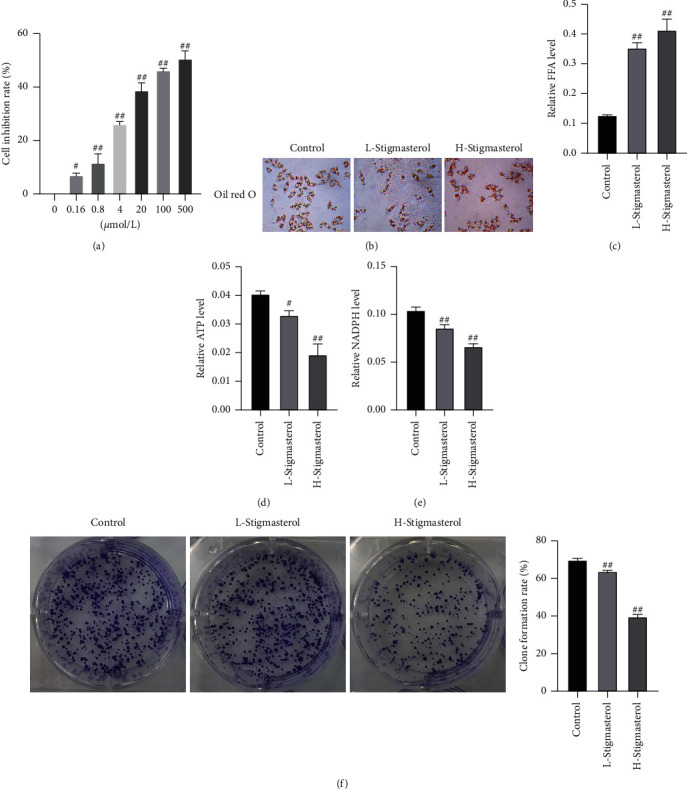
Stigmasterol inhibits the viability and energy metabolism of NCI-H1975 cells. (a) MTT detection. (b) Oil red O staining, magnification of 100x. (c) Free fatty acid detection. (d) ATP detection. (e) NADPH detection. (f) Cell colony formation assay. Compared with the control group, ^#^*p* < 0.05; ^##^*p* < 0.01.

The inhibitory effect of stigmasterol on NCI-H1975 cell viability was detected via MTT, and findings showed that stigmasterol could markedly inhibit the viability of NCI-H1975 cells. As the stigmasterol concentration increased, the inhibition rate of NCI-H1975 cells was improved accordingly (Figure 2(a)).

Subsequently, we selected the 20 and 100 *μ*mol/L stigmasterol as the low-concentration group and high-concentration group, respectively, and continued to clarify the regulatory function of stigmasterol. The regulation of stigmasterol on lipid deposition in NCI-H1975 cells was determined using oil red O staining and free fatty acid detection. The results showed that after stigmasterol treatment, the lipid droplet content in NCI-H1975 cells was significantly increased, the oil red O staining was deepened (Figure 2(b)), and the free fatty acid content was markedly increased. The free fatty acid content in the control group, low stigmasterol concentration group, and high stigmasterol concentration group were 0.12, 0.35, and 0.41 *μ*mol/mg prot, respectively (Figure 2(c)). The above results indicated that stigmasterol promoted fat deposition in NCI-H1975 cells.

We subsequently determined the cell ATP and NADPH contents in the three groups, and the results showed that the ATP contents of control, low-, and high-concentration stigmasterol groups were 0.0404, 0.0331, and 0.0193 *μ*mol/10^6^ cell, respectively (Figure 2(d)). The NADPH contents of the three groups were 0.1040, 0.0855, and 0.0663 *μ*M, respectively (Figure 2(e)). It was shown that after stigmasterol treatment, the intracellular ATP and NADPH contents were reduced, and the reduction effect was more significant with the increase in stigmasterol concentration. Finally, the rate of cell colony formation was detected, which was 69.82, 63.74, and 39.67% in the three groups, respectively (Figure 2(f)). Stigmasterol reduced the energy metabolism of NCI-H1975 cells and affected cell proliferation and the effect of colony formation.

**Figure 3:**
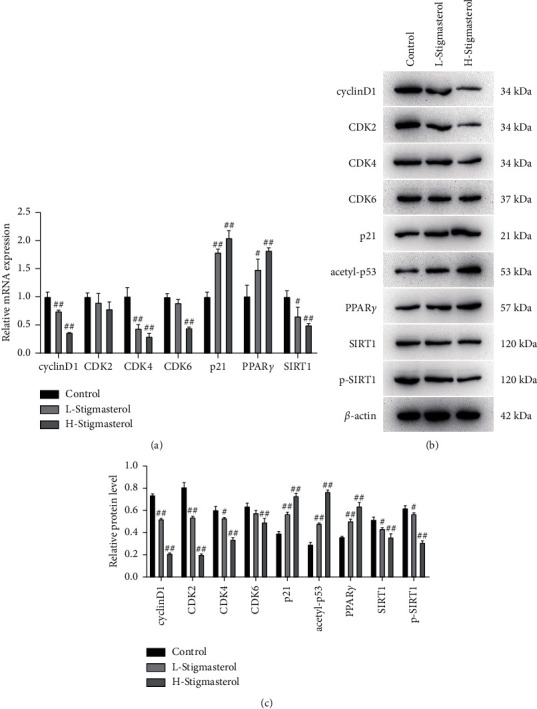
qPCR and western blot assays detects the expressions of cyclins and PPAR*γ*. (a) qPCR detects the expressions of cyclinD1, CDK2, CDK4, CDK6, and p21 and the mRNA expressions of PPAR*γ*-SIRT1 pathway proteins PPAR*γ* and SIRT1. (b–c) Western blot detects the expressions of cyclinD1, CDK2, CDK4, CDK6, p21, and acetyl-p53 and the expressions of PPAR*γ*-SIRT1 pathway proteins PPAR*γ* and SIRT1. Compared with the control group, ^#^*p* < 0.05; ^##^*p* < 0.01.

The mRNA expression changes of cyclins cyclinD1, CDK2, CDK4, CDK6, and p21, and PPAR*γ*-SIRT1 pathway proteins PPAR*γ* and SIRT1 were detected using qPCR assays. The results revealed that expression levels of cyclinD1, CDK4, and SIRT1 in the low-concentration stigmasterol group were greatly inhibited, while p21 and PPAR*γ* were significantly increased as compared with the control group. In the high stigmasterol concentration group, expression levels of cyclinD1, CDK4, CDK6, and SIRT1 were significantly decreased, whereas expressions of p21 and PPAR*γ* were markedly elevated (Figure 3(a)). Western blot detected the expression changes of cyclinD1, CDK2, CDK4, CDK6, p21, and acetyl-p53 as well as the PPAR*γ*-SIRT1 pathway proteins PPAR*γ* and SIRT1. The results indicated a marked decrease in the expressions of cyclinD1, CDK2, CDK4, SIRT1, and p-SIRT1 in the stigmasterol low-concentration group while a decrease in the expression of p21, acetyl-p53, and PPAR*γ* as compared with the control group. In the high-concentration stigmasterol group, the expressions of cyclinD1, CDK2, CDK4, CDK6, SIRT1, and p-SIRT1 were greatly decreased, whereas the expressions of p21, acetyl-p53, and PPAR*γ* were markedly increased (Figures 3(b)and3(c)). The findings indicated that stigmasterol could affect the expression levels of cell cyclins and PPAR*γ*-SIRT1 signaling pathway proteins.

### 3.3. Stigmasterol Inhibits Tumorigenesis of NCI-H1975 Cells in Nude Mice

**Figure 4:**
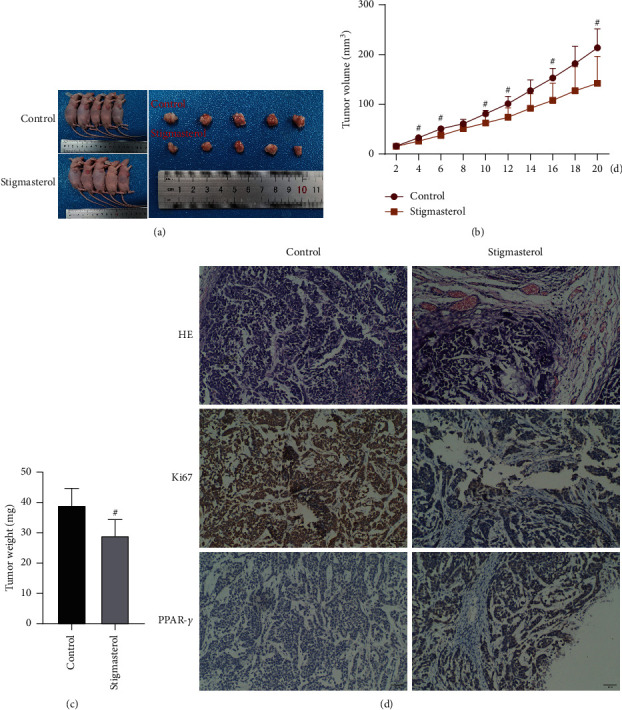
Stigmasterol inhibits tumorigenesis of NCI-H1975 cells in nude mice. (a) Nude mouse tumorigenicity assay, vector, and tumors *in vitro*. (b) Tumor volume records and statistical results. (c) Tumor weight measurement results. (d) HE staining and immunohistochemical staining, magnification of 100x. Compared with the control group, ^#^*p* < 0.05.

Next, the regulatory role of stigmasterol on lung adenocarcinoma was further explored in the animals. After being injected with NCI-H1975 cells, the nude mice of the treatment group were given stigmasterol (40 mg/kg) intragastrically and mice of the control group were given normal saline each day. After 20 days of the administration, the tumor was removed for observation, as shown in Figure 4(a), the tumor size of the stigmasterol group was smaller than the control group with a measurement every 2 d after administration. From day 4, the tumor size of nude mice in the stigmasterol groups was much smaller than the control group (Figure 4(b)). The tumor weight was also measured ultimately, which indicated that the tumor weight in the stigmasterol group was apparently lighter than the control group (Figure 4(c)).

Tissue structure and protein expression of the tumor were detected via HE staining and immunohistochemistry. As compared with the control group, pathological sections of transplanted tumors in nude mice of the stigmasterol group were observed under a microscope, suggesting varying degrees of degeneration in the tumor tissue cells. Large areas of ischemic necrosis appeared in central and marginal cells, especially in areas with abundant blood supply. Residual tumor cells were seen around the necrotic foci. The cells in the control group were vigorous, heteromorphic, and varied in size. The nuclei were large and hyperchromatic. The pathological mitotic phase was revealed free from apparent necrosis areas (Figure 4(d)). Immunohistochemistry results revealed that Ki67 expression in the stigmasterol group was substantially decreased, while PPAR*γ* expression was greatly increased as compared with the control group.

### 3.4. PPAR*γ* Inhibitor can Alleviate the Tumor Suppressor Effect Induced by Stigmasterol

**Figure 5:**
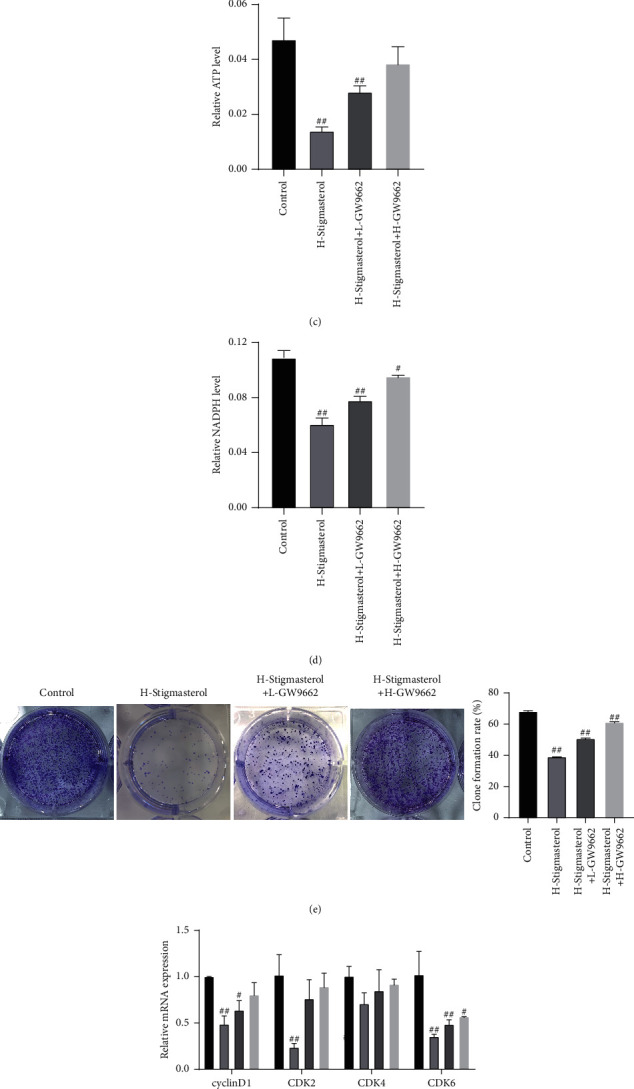
PPAR*γ* inhibitor alleviates the anticancer effect induced by stigmasterol. The cells were co-treated with high concentrations of stigmasterol and GW9662 at 20 and 40 *μ*mol/L, respectively, to detect the regulation of cell viability and metabolism. (a) Oil red staining, magnification of 100x. (b) Free fatty acid detection. (c) ATP detection. (d) NADPH detection. (e) Cell colony formation assay. (f) qPCR was applied to detect the expression of cyclinD, CDK2, CDK4, and CDK6. (g–h) Western blot was applied to detect the expression of cyclinD, CDK2. CDK4, and CDK6. Compared with the control group, ^#^*p* < 0.05; ^##^*p* < 0.01.

Molecular docking predicted that stigmasterol might target PPAR*γ* for regulatory functions. The cells were subsequently co-treated with high-concentration stigmasterol (100 *μ*mol/L) and GW9662 at 20 and 40 *μ*mol/L, respectively, to detect the regulation of cell viability and metabolism. Results of oil red O staining and free fatty acid detection showed that after the addition of GW9662, the lipid droplet content in NCI-H1975 cells was significantly reduced, the color oil red O staining faded (Figure 5(a)), and the free fatty acid content was substantially reduced. The contents of free fatty acid of control group, high-concentration stigmasterol group (100 *μ*mol/L), high-concentration stigmasterol + low-concentration GW9662 group (20 *μ*mol/L), and high-concentration stigmasterol + high-concentration GW9662 group (40 *μ*mol/L) were 0.11, 0.34, 0.30, and 0.25 *μ*mol/mg prot, respectively (Figure 5(b)). The ATP contents of the four groups were 0.0473, 0.0140, 0.0282, and 0.0385 *μ*mol/10^6^ cell, respectively (Figure 5(c)). The cell NADPH contents of the four groups were 0.1133, 0.0565, 0.0818, and 0.0974 *μ*M, respectively (Figure 5(d)). The colony formation rates of the four groups were 68.17, 39.00, 50.70, and 61.18%, respectively (Figure 5(e)). The previously described results indicated that the PPAR*γ* inhibitor GW9662 could mediate the inhibitory effect of stigmasterol on NCI-H1975 cells.

The expression levels of cell cyclins cyclinD1, CDK2, CDK4, and CDK6 were detected using qPCR and western blot assays, implying that stigmasterol could reduce the mRNA and protein expression of cyclinD1, CDK2, CDK4, and CDK6, whereas in the stigmasterol + GW9662 co-treatment group, the mRNA and protein expressions of cyclinD1, CDK2, CDK4, and CDK6 presented an increasing trend (Figures 5(g)and 5(h)).

## 4. Discussion

In recent years, the antitumor efficacy of Chinese medicine has gradually been recognized by researchers at home and abroad. TCM has been proved to reduce the toxicity of chemotherapy drugs, enhance the sensitivity of radiotherapy, minimize the side effects of radiotherapy, and improve the immunity of patients in the treatment of lung cancer [22]. Moreover, it may also have a potential role in anti-recurrence and metastasis [23]. TCM against lung cancer can stabilize lesion conditions, prolong the survival period of patients, and benefit patients with better quality of life. Integrated traditional Chinese and Western medicine is easier to be accepted by patients. Therefore, researchers are devoted to discovering high-efficiency and low-toxicity antitumor drugs through TCM. At present, it is urgently needed to investigate the key monomers of TCM and the regulatory molecular mechanisms. Several research teams, domestic and overseas, have focused on exploring the molecular mechanisms of TCM, which has laid a solid foundation for the extensive and precise application of TCM [24]. To clarify the regulatory effect and molecular mechanism of stigmasterol, a monomer of AhBl on lung adenocarcinoma, this study detected the tumorigenic regulatory effect of stigmasterol on lung adenocarcinoma in human lung adenocarcinoma cells NCI-H1975 cultured *in vitro* and in nude mice. It has been clarified that monomer stigmasterol targeted PPAR*γ* and inhibited the viability and tumorigenicity of lung adenocarcinoma cells NCI-H1975. The present study provided a reference for the molecular mechanism analysis of AhBl and its monome stigmasterol.

AhBl is a widely applied TCM in hospitals in China. Previous studies have shown that 30 patients with non-small cell lung cancer (NSCLC) who had been introduced with AhBl decoction (30–60 g) can stabilize the disease conditions [25]. Compared with the chemotherapy group, the survival rate, lesion stability, and quality of life of the patients were substantially improved, with no apparent toxic and side effects complained, and the effective rate was 67%. Of which, 25 of the 30 cases had half-year survival and 21 had 1-year survival. Jin-Fu-An decoction was introduced to treat NSCLC at middle and advanced stages, including 15 g of AhBl and 15 g of unprocessed *Rhizoma Pinelliae*. Both the TCM group and the integrated traditional Chinese and Western medicine group could benefit patients with better quality of life, implying a significant difference from the Western medicine group (gemcitabine + cisplatin). Unfortunately, TCM did not work well in reducing tumor efficiency. In terms of tumor stability, the integrated traditional Chinese and Western medicine groups presented the most satisfactory effect without a significant difference as compared with the TCM group [25]. Twenty-eight cases with advanced NSCLC were managed with a formula containing AhBl for tumor treatment, and AhBl formula produced equivalent effects to the Western medicine group in terms of clinical efficacy, 1-year survival rate, median survival time, and quality of life were significantly improved. Some researchers have also applied self-prescribed formula (including AhBl 30 g) to treat advanced NSCLC. After three to four courses of drug administration in comparison to paclitaxel combined with cisplatin chemotherapy, the curative effect and median survival time of both therapies tended to be the same. The TCM group was higher than the chemotherapy group in terms of adverse reactions, tolerance, and improvement of cancer patients' quality of life. It can be seen that, in the empirical formula of TCM, AhBl has been widely applied against lung cancer in the clinic, but its molecular regulation mechanism has been rarely reported [26].

As for the research on stigmasterol, the main monomer of AhBl, there are some reports at home and abroad. In a study using Swiss albino mice, the antitumor effect of stigmasterol on Ehrlich ascites carcinoma has been examined, in which stigmasterol has been intraperitoneally injected into mice with ascites cancer at doses of 5 and 10 mg/kg, respectively. As compared with the blank control, the inhibition rates of stigmasterol on tumor cells were 49.92 and 60.11%, respectively. The tumor weight inhibition rates were 60.52 and 83.63%, respectively. The mean survival time of the treatment groups increased from 16.15 d to 33.7 d and 37.48 d, respectively. Furthermore, additional studies have also found that the inhibitory effect of stigmasterol on SMMC-7721 is dose- and time-dependent, it can significantly downregulate various oncogenes fos, myc, and ras, and upregulate the expressions of tumor suppressor gene and phosphokinase Map2k6 represented by NF-2 [27]. Subsequent studies on its action mechanism have discovered that stigmasterol in *Hedyotis diffusa* had a certain inhibitory effect on liver cancer cells *in vitro* and *in vivo*. It promotes apoptosis of hepatocellular carcinoma H22 through regulating the cell cycles, upregulating Map2k6 expression, and downregulating Bcl-2 gene expression in hepatoma cells [28]. The study of antitumor activity of stigmasterol and reactive oxygen species (ROS) has revealed that stigmasterol induces apoptosis in human hepatoma cell SMMC-7721 through ROS oxidation, the cascade reaction of increased calcium ion concentration, and the arrest of cell cycles. Stigmasterol can upregulate the expressions of pro-apoptotic genes (Bax protein and p53 gene), downregulate Bcl-2 gene expression, and activate caspase-8 and -9 protein expression. And it induces apoptosis of liver cancer cells by damaging cellular DNA [27]. In the present study, our research findings revealed that the addition of stigmasterol markedly inhibited the viability of NCI-H1975 cells but promoted lipid deposition of NCI-H1975 cells. Meanwhile, the reduction of cell energy metabolism affected cell proliferation and colony formation. qPCR and western blot assays indicated that stigmasterol could regulate the levels of cyclins and PPAR*γ* signaling pathway proteins. Taken together, this study indicated that stigmasterol could regulate cell cyclins and affect the cell cycles, thereby regulating the metabolic activity and proliferation ability of cells.

Additionally, the present research also found that stigmasterol had a strong binding effect on PPAR*γ* protein. Previous studies have shown that PPAR*γ* expression in tumor tissues of NSCLC is higher than that in surrounding normal tissues. And they argue that the reason for the high expression of PPAR*γ* protein in NSCLC may be the lack of PPAR*γ* endogenous ligands in tumor tissue. PPAR*γ* protein cannot be decomposed if it is not activated by a ligand, which accumulates abnormally in tumor cells [29]. Additional studies have indicated that PPAR*γ* is expressed in the transplanted tumors of Lewis lung cancer mice, and GW9662 can significantly inhibit its expression. Artemether can substantially upregulate the expression of PPAR*γ*, suggesting that it can activate abnormally accumulated PPAR*γ* in tumor tissue [30]. The assays of nude mouse tumorigenesis suggested that both tumor volume and tumor weight of the stigmasterol-treated group were apparently lower as compared with the control group. Tumor tissue cells developed varying degrees of degeneration and large areas of ischemic necrosis presented in the central and peripheral cells. Immunohistochemistry results revealed that, compared with the control group, Ki67 expression in the stigmasterol group was substantially reduced, whereas PPAR*γ* expression was greatly elevated. The PPAR*γ* inhibitor GW9662 could mediate the inhibitory effect of stigmasterol on NCI-H1975 cells.

To the best of our knowledge, our study for the first time illustrated that stigmasterol is the main active component of AhBl and it could target PPAR*γ* to exert effects on inhibiting tumorigenesis and tumor growth of lung adenocarcinoma, providing an experimental basis for stigmasterol as a therapeutic for cancer treatment. And based on the current literature and this study, we suggest that stigmasterol may be used in combination with Western medicines and PPAR*γ* agonists to maximize its effectiveness. However, this study also has some limitations. The regulatory function of PPAR*γ* inhibitor GW9662 was not verified at the animal level. In addition, this study failed to further verify whether stigmasterol could play a regulatory function by constructing a PPAR*γ* knockout cell model or animal model. AhB1 and its monomer stigmasterol are still worthy to be studied, their anti-carcinogenic potential will bring welfare to cancer patients. In the future, we will continue to investigate the molecular regulation mechanism of AhBl and stigmasterol based on this research to provide a research basis for better application of Chinese medicinal herbs.

## 5. Conclusions

Stigmasterol inhibited the viability of NCI-H1975 cells, promoted cell lipid deposition, reduced cell energy metabolism, and impaired cell proliferation and colony formation. It could also markedly inhibit tumorigenesis of NCI-H1975 cells in nude mice. The PPAR*γ* inhibitor GW9662 could mediate the inhibitory effect of stigmasterol on NCI-H1975 cells.

## Figures and Tables

**Table 1:** Sequences of qPCR primers.

Gene name	Primer sequence (5′ to 3′)
cyclinD1	Forward CACGGCTCACGCTTACCTCA
	Reverse ACTTGCGCGTCACAGGACAG
CDK2	Forward CCAGGAGTTACTTCTATGCCTGA
	Reverse TTCATCCAGGGGAGGTACAAC
CDK4	Forward ATGGCTACCTCTCGATATGAGC
	Reverse CATTGGGGACTCTCACACTCT
CDK6	Forward GCTGACCAGCAGTACGAATG
	Reverse GCACACATCAAACAACCTGACC
p21	Forward GACTGTGATGCGCTAATGG
	Reverse TTCCTGTGGGCGGATTAG
PPAR*γ*	Forward TCTCTCCGTAATGGAAGACC
	Reverse GCATTATGAGACATCCCCAC
SIRT1	Forward TAGCCTTGTCAGATAAGGAAGGA
	Reverse ACAGCTTCACAGTCAACTTTGT
GAPDH	Forward CTGGGCTACACTGAGCACC
	Reverse AAGTGGTCGTTGAGGGCAATG

## Data Availability

The data used to support the findings of this study are included within the article.

## Additional Points

This study contributes to the field of TCM and oncology. We found AhBl inhibited the growth and tumorigenicity of lung adenocarcinoma cells NCI-H1975, which may be a potential medicine in antitumor.
